# Clinical outcomes of pharmacological therapies for heart failure in Black vs. White populations: a meta-analysis of randomized controlled trials of heart failure treatment

**DOI:** 10.3389/fcvm.2025.1482311

**Published:** 2025-06-30

**Authors:** Yujia Li, Huilin Tang, Wenxi Huang, Wei-Han Chen, Shao-Hsuan Chang, Jiang Bian, Mustafa M. Ahmed, Stephen E. Kimmel, Jingchuan Guo

**Affiliations:** ^1^ Department of Pharmaceutical Outcomes and Policy, University of Florida College of Pharmacy, Gainesville, FL, United States; ^2^ Department of Health Outcomes and Biomedical Informatics, University of Florida College of Medicine, Gainesville, FL, United States; ^3^ Division of Cardiovascular Medicine, University of Florida College of Medicine, Gainesville, FL, United States; ^4^ Department of Epidemiology, University of Florida College of Public Health and Health Professions and College of Medicine, Gainesville, FL, United States

**Keywords:** meta-analysis, heart failure, treatment outcome, racial difference, SGLT2 inhibitors

## Abstract

**Objective:**

To evaluate the effect of different pharmacological therapies for heart failure (HF) between the Black vs. White population.

**Method:**

We included randomized controlled trials (RCT) of HF pharmacological therapies with explicit strata of Black or White adults in the primary or secondary analysis. We examined three outcomes: (1) the composite of CV death or hospitalization for heart failure (HHF), (2) HHF, and (3) all-cause death. Within each race (White and Black), we calculated the pooled risk ratio (RR) with a 95% confidence interval (CI) of different pharmacological therapies using random-effects models. Within each pharmacological therapies, we assess the differences in the treatment effect by race.

**Results:**

In 19 RCT reporting eight pharmacological therapies, there was no significant difference between the Black and White groups for using sacubitril/valsartan, angiotensin-converting enzyme inhibitors, calcium-channel blockers, direct renin inhibitors, oral soluble guanylate cyclase, or vasodilators. Sodium-glucose cotransporter-2 inhibitors (SGLT2i) had a different effect in HHF across the White and Black patients (P_interaction_ = .030), with a better treatment effect observed in the Black (RR 0.39, 95% CI 0.19–0.80) compared to the White group (0.90, 0.71–1.14). Beta-blockers had a better treatment effect in the White (0.65, 0.52–0.81) compared to the Black group (1.14, 0.88–1.47) regarding the all-cause death outcome (P_interaction_ = .001).

**Conclusion:**

Black individuals with HF appeared to obtain a greater benefit of HHF risk reduction from SGLT2i and less benefit for mortality from beta-blockers compared to their White counterparts.

## Introduction

Heart Failure (HF) remains a leading cause of death and hospitalization worldwide (1). Among the various populations affected by HF, Black individuals face a disproportionately high prevalence of HF in comparison to other racial groups (2). In the absence of pre-existing cardiovascular disease, Black individuals are at greater risk of developing HF than other populations (3). The increased incidence can be attributed to complex factors, including genetic disposition (4), cardiometabolic and physiological factors such as relative natriuretic peptides deficiency (5, 6) and higher salt sensitivity (7). Additionally, Black individuals face a higher prevalence of comorbidities such as hypertension and diabetes (2, 8) which further contribute to the onset and progression of HF (9). These differences are reflected in clinical outcomes. Black men have the highest age-adjusted death rates from HF, followed by non-Hispanic White men, Black women, and non-Hispanic White women (118.2, 111.3, 86.0 and 80.4 per 100,000 person-years, respectively) (10). The rate of HF hospitalization is nearly 2.5 times higher in Black patients than in White patients, and Black individuals also experience longer hospital stays and a higher 90-day readmission rate (9). Despite their elevated risk of clinical outcomes, Black patients have been vastly underrepresented in randomized controlled trials (RCT) evaluating HF therapies (11). It remains unclear whether HF medications are comparably effective in Black and White populations. To address this knowledge gap, we conducted a meta-analysis of RCT to evaluate differences in the effect of HF pharmacotherapies between Black and White populations.

## Method

This meta-analysis was conducted according to the Preferred Reporting Items for Systematic Reviews and Meta-Analyses (PRISMA) statement. We systematically queried Medline, Embase, and the Cochrane Central Register of Controlled Trials (CENTRAL) databases from inception until February 9, 2022, to identify eligible RCT. Additionally, we manually queried reference lists of included RCT, relevant meta-analyses, and any other published trials since February 9, 2022, to identify other potential trials. Three reviewers (WH, WC, and SC) independently selected studies based on following inclusion criteria: (1) RCT in adults (aged ≥18 years) with a diagnosis of HF; (2) trial with explicit strata of Black or White adults in primary or secondary analysis; (3) trial comparing one pharmacotherapy of interest with another pharmacotherapy or placebo/no use; (4) trials reporting at least one of the following outcome, including all-cause death, hospitalization for heart failure (HHF), and the composite endpoint of cardiovascular (CV) death or HHF; and (5) trials with a sample size ≥100. The pharmacotherapy of interest included angiotensin-converting enzyme inhibitors (ACEi), angiotensin receptor blockers, angiotensin receptor-neprilysin inhibitors (ARNi), beta-blockers, calcium-channel blockers, direct renin inhibitors, oral soluble guanylate cyclase stimulator, sodium-glucose cotransporter-2 inhibitors (SGLT2i), vasopressin V2 receptor blockers and vasodilators. We examined three outcomes: all-cause death, HHF, and the composite of CV death or HHF.

Data extraction was independently conducted by four reviewers (WH, WC, SC, and YL) using a standardized form. Reviewers worked in pairs, with each pair cross-checking the extracted data for accuracy. Extracted data included study-level characteristics (first author, publication year, NCT number, and duration of follow-up) and characteristics of patients (inclusion criteria, mean age, and proportion of male participants). Study quality was assessed independently by two groups of reviewers, with two reviewers in a group using the Cochrane Risk of Bias tool (12). Risk of bias was evaluated across seven domains: random sequence generation, allocation concealment, blinding of participants and personnel, blinding of outcome assessment, incomplete outcome data (attrition bias), selective outcome reporting (reporting bias), and other bias. For each domain, studies were rated as having low, high, or unclear risk of bias. Any discrepancies between groups were resolved through discussion and consensus, and when necessary, a third reviewer was consulted to adjudicate unresolved differences.

Within each racial group (White and Black), we assessed the effect of each pharmacotherapy compared with the control group on the risk of outcomes. We calculated pooled risk ratio (RR) and 95% confidence interval (CI) using random-effect models. We evaluated the heterogeneity between studies using the *I*^2^ statistic. Within each drug class, we assessed the differences in the treatment effect by race. This study was considered exempt from review by the University of Florida Institutional Review Board. All statistical analyses were performed using STATA (version 17, Stata Corp., College Station, TX). A *p*-value of less than 0.05 indicated statistical significance.

## Results

**Figure 1:**
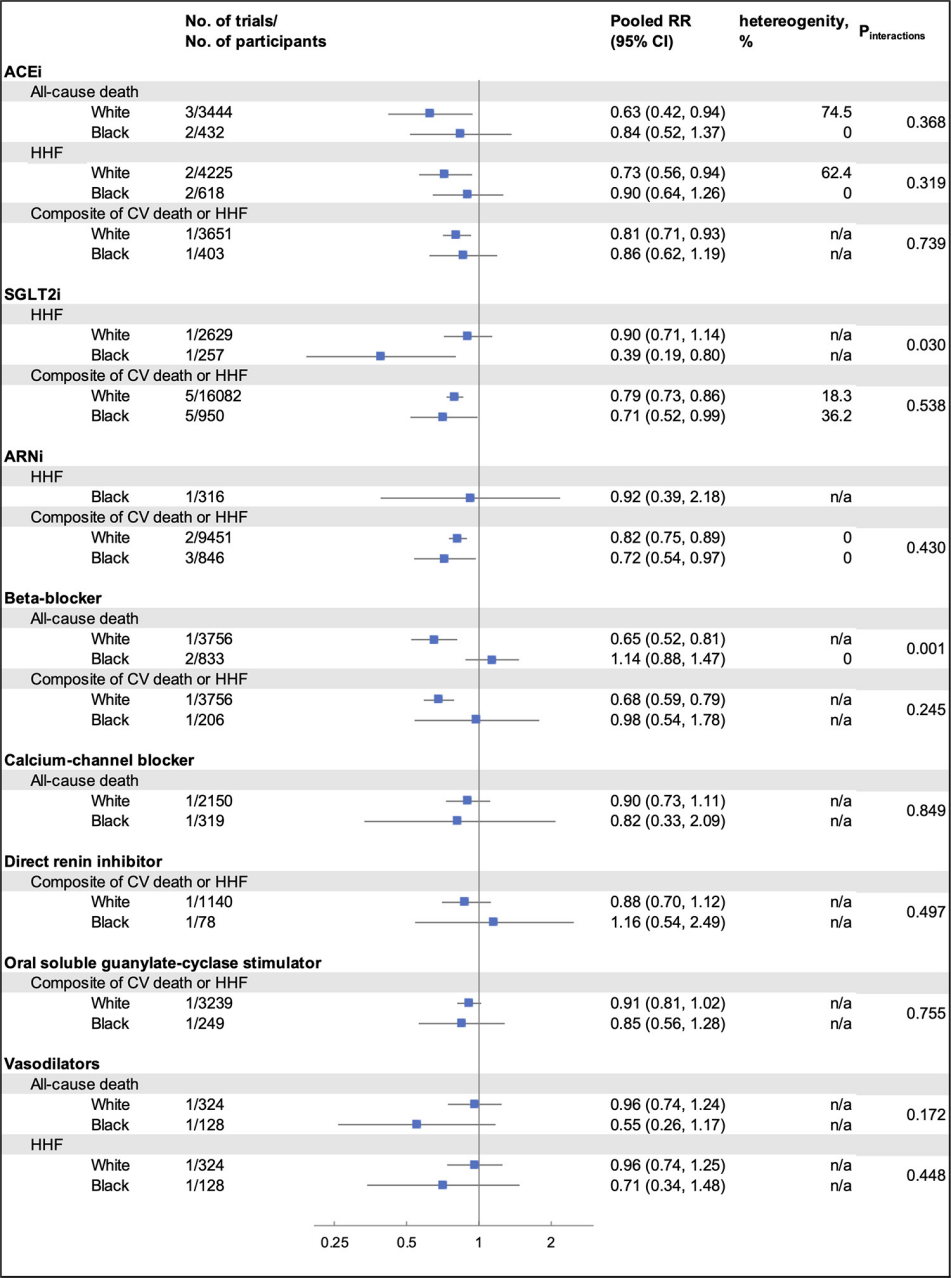
Summarized results from the meta-analysis, stratified by pharmacotherapy, outcomes, and race. RR, relative risk; CI, confidence interval; ACEi, angiotensin-converting enzyme inhibitor; HHF, hospitalization for heart failure; CV, cardiovascular; SGLT2i, sodium–glucose cotransporter 2 inhibitor; ARNi, angiotensin receptor–neprilysin inhibitor; Between-subgroup heterogeneity (*I*^2^) of each pharmacotherapy was calculated based on the random effects model within each racial groups and by outcomes; n/a indicates that the heterogeneity could not be tested due to limited eligible trials. For ACEi in all-cause death, one trial (SAVE, 1992) reported results from White and non-White subgroup. For ARNi in HHF outcome and the composite outcome of CV death or HHF, one trial (PIONEER-HF, NCT02554890, 2018) reported results from Black and non-Black subgroup. Thus, results from Black subgroup for both outcomes were included in our analysis. For beta-blockers in all-cause death outcome, one trial (BEST, 2001) reported results of Black and non-Black subgroup. Results from only Black subgroup were included in our analysis.

A total of 16,362 citations were retrieved through our electronic search. After screening the titles/abstracts and full texts based on the inclusion and exclusion criteria, we included 19 RCT reporting eight pharmacotherapies for HF, including 40,287 participants. The PRISMA flow diagram showing the process of identifying and selecting eligible trials is presented in Supplementary Figure S1. Table 1 shows the clinical characteristics of the included studies. The average sample size across trials was 2,120 (range: 642–6,263), with Black participants comprising 8.6% (3,489 out of 40,287) of the total study population. The mean duration of follow-up was 20.7 months, ranging from 2–42 months). The risk of bias assessment for the 19 trials included in this meta-analysis generally showed a low to unclear risk across most domains (Supplementary Table S5). The summary results are presented in Figure 1. Among White individuals, there was no significant difference in the overall rate of HHF (*I*^2^ = 58.7%, *p* = 0.28) across different pharmacotherapies. However, there is significant heterogeneity in the rate of all-cause death (*I*^2^ = 68.1%, *p* = 0.03) and the composite of CV death or HHF (*I*^2^ = 66.6%, *p* < 0.01). In Black individuals, there was no significant difference in the overall rate of all-cause death (*I*^2^ = 13.4%, *p* = 0.22), HHF (*I*^2^ = 9.0%, *p* = 0.26), or the composite of CV death or HHF (*I*^2^ = 4.0%, *p* = 0.76) in using different pharmacotherapies. When comparing the effect of each pharmacotherapy by race, SGLT2i had a different effect in HHF across White and Black patients (p_interaction_ = 0.03), with a better treatment effect observed in Black patients (RR 0.39; 95% CI 0.19–0.80) compared to White patients (RR 0.90; 95% CI 0.71–1.14). Beta-blockers had a greater treatment effect in White patients (RR 0.65; 95% CI 0.52–0.81) than Black patients (RR 1.14; 95% CI 0.88–1.47) regarding all-cause death (p_interaction_ < 0.01).

**Table 1:** Characteristics of the included randomized controlled trials.

First author year (Trial Name)	Intervention	Control	Population	No. of total participants	No. of White Patients	No. of Black patients	Age, yrs	Male, %	Mean/Median follow-up, months
Solomon 2022 (DELIVER)	Dapagliflozin/SGLT2i	Placebo	Adults ≥ 40 years, with or without diabetes, with stabilized HF and LVEF ≥ 40%	6,263	4,439	159	71.6	56.2	27.6
Bhatt 2021 (SOLOIST-WHF)	Sotagliflozin/SGLT2i	Placebo	Adults 18–85 years, with T2DM and hospitalization for HF	1,222	567	25	69.5	66.2	9
Anker 2021 (EMPEROR-Preserved)	Empagliflozin/SGLT2i	Placebo	Adults ≥ 18 years with heart failure and LVEF > 40%	5,988	2,286	133	71.8	55.4	26.2
Packer 2020, Lam 2021 (EMPEROR-Reduced)	Empagliflozin/SGLT2i	Placebo	Adults ≥ 18 years with heart failure and LVEF ≤ 40%	3,730	1,325	123	66.8	76.1	16
Armstrong 2020 (VICTORIA)	Vericiguat/oral soluble guanylate cyclase stimulator	Placebo	Adults ≥ 18 years with worsening heart failure and LVEF ≤ 45%	5,050	3,239	249	67.3	76.1	10.8
McMurray 2019 (DAPA-HF)	Dapagliflozin/SGLT2i	Placebo	Adults ≥ 18 years with heart failure and LVEF < 40%	4,744	1,662	122	66.4	76.6	18.3
Solomon 2019 (PARAGON-HF)	Sacubitril-valsartan/ARNi	Valsartan/CCB	Adults ≥ 50 years, with heart failure and LVEF ≥ 45%	4,822	3,907	102	72.7	48.3	35
Morrow 2019, Berardi 2020 (PIONEER-HF)	Sacubitril-valsartan/ARNi	Enalapril/ACEi	Adults hospitalized for acute decompensated heart failure with reduced ejection fraction	881	515	316	62.0	72.1	2
McMurray 2014 (PARADIGM-HF)	LCZ696/ARNi	Enalapril/ACEi	Adults ≥ 18 years with heart failure and LVEF ≤ 35%	8,442	2,763	213	64	78.3	27
Gheorghiade 2013 (ASTRONAUT)	Aliskiren/Direct renin inhibitor	Placebo	Adults ≥ 18 years with heart failure and LVEF ≤ 40%	1,615	575	36	64.6	77.2	11.3
Konstam 2007 (EVEREST)	Tolvaptan/Diuretics	Placebo	Adults ≥ 18 years with heart failure and reduced ejection fraction (LVEF ≤ 40%)	4,133	1,767	NA	65.8	74.0	9.9
BEST investigators, 2001 (BEST)	Bucindolol/beta-blocker	Placebo	Adults with heart failure and LVEF ≤ 35%	2,708	1,896	627	60.0	78.0	24
Levine 2000 (MACH-1)	Mibefradil/CCB	Placebo	Adults with heart failure and LVEF < 35%	2,590	1,070	164	62.8	79.4	20
MERIT-HF Study group 1999, Goldstein 2003 (MERIT-HF)	Metoprolol CR/XL/beta blocker	Placebo	Adults with heart failure and LVEF ≤ 40%	3,991	3,756	208	NR	77.5	12
Packer 1996, Yancy 2001 (U.S. Carvedilol HF)	Carvediol/ACEi	Placebo	Adults with heart failure and LVEF < 35%	1,094	877	217	57.9	76.6	6.5
Pfeffer 1992, Moye 1994 (SAVE)	Cartopril/ACEi	Placebo	Adults 21–80 years with heart failure and LVEF ≤ 40%	2,231	1,993	NA	59.4	82.4	42
The SOLVD Investigators 1992, Dries 1999 (SOLVD)	Enapril/ACEi	Placebo	Adults with heart failure and LVEF ≤ 35%	4,228	3,658	404	59.1	88.6	37.6
Cohn 1991, Carson 1999 (V-HeFT II)	Enapril/ACEi	Hydralazine-isosobide dinitrate/vasodilators	Adults 18–75 years with heart failure and LVEF < 45%	804	574	230	60.6	100	30
Cohn 1986, Carson 1999 (V-HeFT)	Hydralazine-isosobide dinitrate/vasodilators	Prazosin or placebo	Adults 18–75 years with heart failure and LVEF < 45%	642	450	180	58.3	100	27.6

SGLT2i, sodium–glucose cotransporter 2 inhibitor; HF, heart failure; LVEF, left ventricular ejection fraction; T2DM, type 2 diabetes mellitus; ARNi, angiotensin receptor–neprilysin inhibitor; CCB, calcium channel blocker; ACEi, angiotensin-converting enzyme inhibitor.

## Discussion

The present meta-analysis compared the efficacy of pharmacotherapies in patients with HF between White and Black populations. While significant reductions in the risk of the composite of CV death or HHF were observed in both White and Black participants for most pharmacotherapies, we identified some differences in treatment effects by race.

Black individuals appeared to derive greater benefit from SGLT2i in reducing the risk of HHF compared to White individuals, with a statistically significant treatment-by-race interaction observed. This heterogeneity may reflect underlying differences in cardiometabolic profiles, including variations in glucose homeostasis and cardiovascular function (13). Additionally, SGLT2i-induced natriuresis may be particularly beneficial for Black patients, who often have deprivation of natriuretic peptides, potentially leading to more pronounced reductions in plasma volume and blood pressure (14, 15). While this interaction was derived from findings of a single trial with limited representation of Black participants and may be due in part to chance, it is notable that Black participants also demonstrated a larger reduction in risk, though not significant, in the composite outcome of CV death or HHF. The benefit observed in a historically underrepresented population is compelling and further mechanistic study is needed to clarify the biological pathway that drives the potential differential responses to SGLT2i across racial groups.

Conversely, beta-blockers were associated with an increased risk of all-cause death among Black patients compared to White patients. The differential effect was largely driven by the findings from the Beta-Blocker Evaluation of Survival (BEST) trial, which reported a nominally significant interaction indicating increased mortality among Black participants. Importantly, the Black participants in BEST had more advanced heart failure and lower ejection fractions (16), placing them at greater baseline risk. Additionally, the study drug bucindolol also has a potent sympatholytic effect, which might lead to a more pronounced reduction in norepinephrine among Black patients, contributing to adverse outcomes (17). Nevertheless, these findings are largely consistent with retrospective observational studies that suggested diminished protective effects of beta-blockers in Black populations (18). The observed difference can be explained by a combination of genetic and physiological factors. Genetic polymorphisms, particularly the Arg389Gly polymorphism in the β1-adrenergic receptor gene (ADRB1), which has been shown to be associated with decreased beta-blocker efficacy (19), are more prevalent in Black individuals (20). Additionally, Black patients often exhibit lower renin levels and cardiac output, with increased peripheral resistance, which may reduce beta-blocker responsiveness (21, 22). Nevertheless, these results underscore the importance of considering race as a factor in treatment decisions and the potential need for more personalized approaches to heart failure management.

To our knowledge, this is one of the first meta-analyses to systematically examine clinical outcomes of HF pharmacotherapies across multiple drug classes in Black vs. White populations. While previous meta-analyses and systematic reviews have noted potential racial differences in response to HF treatments, they have largely relied on older trials with traditional agents (17, 23). Our analysis builds on the literature by incorporating more recent therapies, including ARNi and SGLT2i. Our results suggest that these newer pharmacologic treatments are beneficial in Black populations, even when effect sizes differ modestly from those observed in White populations. By synthesizing trial-based evidence across diverse populations, our findings contribute to the evidence base that may be considered in future evaluations of guideline recommendations, and may inform discussions about equitable treatment access and the importance of inclusivity in trial design.

Our results should be interpreted in light of several limitations. First, the subgroup analyses reported by these trials were not truly randomized, and we had no access to detailed data from individual trials by race, which precluded more granular or adjusted analysis. Second, our reliance on broad racial categories of “Black” and “White”, as reported in each RCT, may oversimplify the diverse genetic backgrounds and lived experiences within these populations. Third, only a number of trials were eligible for inclusion in the current meta-analyses that reported prespecified outcomes stratified by race, and most drug–outcome–race strata included only 3–4 trials. This limited sample size constrained our ability to formally assess publication bias, as the sample in each stratum is below the recommended threshold (≥10 studies) for reliable detection (24). As a result, we cannot rule out the possibility that smaller or negative studies may be underrepresented in the literature. Additionally, substantial heterogeneity was observed in certain comparisons, and due to limited studies within each stratum, we did not formally test for sources of variability. Therefore, our pooled estimates should be interpreted with caution, particularly where differences in underlying population or treatment between studies may contribute to observed heterogeneity. Finally, we did not perform separate analyses for HF patients with reduced vs. preserved ejection fraction by each race. HF patients with reduced or preserved ejection fracture due to the limited eligible study included by each race. This distinction is crucial, as these two types of heart failure have different underlying pathophysiology and potentially different treatment responses across racial groups. Future research should focus on distinguishing such patients to better understand how patients of different races and types of HF could respond to different pharmacotherapies.

In summary, compared with White individuals, Black individuals with HF appeared to obtain a greater benefit of reduced HHF risk with SGLT2i. Our data support the prioritization of SGLT2i among Black individuals with HF to improve long-term health outcomes. However, due to the nature and limitations of the meta-analysis, further research in real-world settings is needed to confirm our findings.

## Data Availability

The data supporting the conclusion of this article will be made available by the authors, without undue reservation.
