# Supplementary material for: Clinical outcomes of pharmacological therapies for heart failure in Black vs. White populations: a meta-analysis of randomized controlled trials of heart failure treatment
Source: Front Cardiovasc Med. 2025 Jun 30;12:1482311. doi: 10.3389/fcvm.2025.1482311 (PMC12256505; doi:10.3389/fcvm.2025.1482311)
Supplement: Supplementary file 1 [file Datasheet1.docx]

**SUPPLEMENTAL MATERIALS**

**Supplementary Table 1.** Search strategy in Ovid Medline (Date of search: Feb 22, 2022)

| **#** | **Query** |
| --- | --- |
| 1 | exp Heart Failure/ |
| 2 | Cardiomyopathy, Dilated/ |
| 3 | (heart failure or cardiac failure or cardiac insufficiency or cardiomyopath$).tw. |
| 4 | ((cardi$ or myocard$) adj2 (failure$ or insufficien$)).tw. |
| 5 | or/1-4 |
| 6 | exp angiotensin receptor-neprilysin inhibitor/ or ARNI.mp. |
| 7 | (((((LCZ696 or LCZ 696 or LCZ-696 or valsartan) adj6 sacubitril) or valsartan) adj6 sacubitril) or valsartan sacubitril or valsartansacubitril or sacubitril adj6valsartan or sacubitril valsartan or sacubitril-valsartan).mp. |
| 8 | exp dipeptidyl carboxypeptidase inhibitor/ or exp Angiotensin-Converting Enzyme Inhibitors/ |
| 9 | (angiotensin converting enzyme inhibitor or ACEI or ACEI or antagonist$ or inhibitor$ benazepril or captopril or enalapril or fosinopril or imidapril or lisinopril or moexipril or perindopril or quinapril or ramipril or trandolapril or zofenopril or alacepril or cilazapril or spirapril or delapril).mp. |
| 10 | exp beta adrenergic receptor blocking agent/ or exp Adrenergic beta-Antagonists/ |
| 11 | (beta blocker$ or BB or acebutolol or atenolol or betaxolol or bisoprolol or carvedilol or labetalol or metoprolol or nadolol or nebivolol or penbutolol or pindolol or propranolol or sotalol or timolol).mp. |
| 12 | exp aldosterone antagonist/ |
| 13 | (aldosterone antagonist$ or mineralocorticoid-receptor antagonist or MRA or eplerenone or spironolactone or antisterone or aldactone).mp. |
| 14 | exp angiotensin receptor antagonist/ |
| 15 | (angiotensin receptor blocker$ or angiotensin receptor antagonist$ or ARB or azilsartan or candesartan or eprosartan or irbesartan or losartan or olmesartan or telmisartan or valsartan).mp. |
| 16 | (((((sodium-glucose co-transporter 2 or SGLT2 or SGLT2 inhibitor* or sodium glucose) adj6 inhibitor*) or SGLT2 inhibitor* or sodium-glucose) adj6 inhibitor*) or Sodium-Glucose Transporter 2 or sodium glucose-cotransporter 2 or sodium-glucose cotransporter$ or sodium glucose-cotransporter$).mp. |
| 17 | (dapagliflozin or empagliflozin or canagliflozin).mp. |
| 18 | exp ivabradine plus metoprolol/ or exp ivabradine/ or exp carvedilol plus ivabradine/ |
| 19 | (Omecamtiv mecarbil or CK-1827452 or Omecamtiv or mecarbil).mp. |
| 20 | (Vericiguat or guanylate cyclase stimulator or soluble guanylate cyclase stimulator).mp. |
| 21 | (((((Hydralazine-Isosorbide Dinitrate or Hydralazine-Isosorbide) adj6 Dinitrate) or Hydralazine Isosorbide Dinitrate or Hydralazine) adj6 Isosorbide adj6 Dinitrate) or vasodilators).mp. |
| 22 | digoxin.mp. or Digoxin/ |
| 23 | exp phosphodiesterase-5 inhibitor/ |
| 24 | (sidenafil or tadalafil).mp. |
| 25 | (diuretic or loop diuretic).mp. |
| 26 | (furosemide or bumetanide or torasemide or azosemide or eplerenone or hydroclorotiazides).mp. |
| 27 | or/6-26 |
| 28 | "randomized controlled trial".pt. |
| 29 | (random$ or placebo$ or single blind$ or double blind$ or triple blind$).ti,ab. |
| 30 | (retraction of publication or retracted publication).pt. |
| 31 | or/28-30 |
| 32 | (animals not humans).sh. |
| 33 | ((comment or editorial or meta-analysis or practice-guideline or review or letter or journal correspondence) not "randomized controlled trial").pt. |
| 34 | (random sampl$ or random digit$ or random effect$ or random survey or random regression).ti,ab. not "randomized controlled trial".pt. |
| 35 | 32 or 33 or 34 |
| 36 | 31 not 35 |
| 37 | (random$ or placebo$ or single blind$ or double blind$ or triple blind$).ti,ab. |
| 38 | RETRACTED ARTICLE/ |
| 39 | or/37-38 |
| 40 | (animal$ not human$).sh,hw. |
| 41 | (book or conference paper or editorial or letter or review).pt. not exp randomized controlled trial/ |
| 42 | (random sampl$ or random digit$ or random effect$ or random survey or random regression).ti,ab. not exp randomized controlled trial/ |
| 43 | or/40-42 |
| 44 | 39 not 43 |
| 45 | 36 or 44 |
| 46 | 5 and 27 and 45 |

**Supplementary Table 2.** Search strategy in Embase (Date of search: Feb 22, 2022)

| **#** | **Query** |
| --- | --- |
| 1 | ‘Heart Failure’/exp |
| 3 | (‘heart failure’ or ‘cardiac failure’ or ‘cardiac insufficiency’ or ‘cardiomyopath*’):ab,ti,kw |
| 4 | ((cardi* or myocard*) NEAR/2 (failure* or insufficien*)):ab,ti,kw |
| 5 | or/1-4 |
| 6 | ‘angiotensin receptor-neprilysin inhibitor’/exp or ARNI:ab,ti,kw |
| 7 | (‘LCZ696’ OR ‘LCZ 696’ OR ‘LCZ-696’ OR ‘valsartan NEAR/6 sacubitril’ OR ‘valsartan NEAR/6 sacubitril’ OR ‘valsartan sacubitril’ OR ‘valsartansacubitril’ OR ‘sacubitril NEAR/6 valsartan’ OR ‘sacubitril valsartan’ OR ‘sacubitril-valsartan’):ab,ti,kw |
| 8 | ‘dipeptidyl carboxypeptidase inhibitor’/exp or ‘Angiotensin-Converting Enzyme Inhibitors’/exp |
| 9 | (‘angiotensin converting enzyme inhibitor’ or ACEI or ACEI or benazepril or captopril or enalapril or fosinopril or imidapril or lisinopril or moexipril or perindopril or quinapril or ramipril or trandolapril or zofenopril or alacepril or cilazapril or spirapril or delapril):ab,ti,kw |
| 10 | ‘beta adrenergic receptor blocking agent’/exp |
| 11 | (beta blocker* or BB or acebutolol or atenolol or betaxolol or bisoprolol or carvedilol or labetalol or metoprolol or nadolol or nebivolol or penbutolol or pindolol or propranolol or sotalol or timolol):ab,ti,kw |
| 12 | ‘aldosterone antagonist’/exp |
| 13 | (‘aldosterone antagonist*’ or ‘mineralocorticoid-receptor antagonist’ or MRA or eplerenone or spironolactone or antisterone or aldactone):ab,ti,kw |
| 14 | ‘angiotensin receptor antagonist’/exp |
| 15 | (‘angiotensin receptor blocker*’ or ‘angiotensin receptor antagonist*’ or ARB or azilsartan or candesartan or eprosartan or irbesartan or losartan or olmesartan or telmisartan or valsartan):ab,ti,kw |
| 16 | ‘ivabradine plus metoprolol’/exp or ivabradine/exp or ‘carvedilol plus ivabradine’/exp |
| 17 | (‘Omecamtiv mecarbil’ OR ‘CK-1827452’ OR Omecamtiv OR mecarbil):ab,ti,kw |
| 18 | (Vericiguat OR ‘guanylate cyclase stimulator’ OR ‘soluble guanylate cyclase stimulator’):ab,ti,kw |
| 19 | (‘Hydralazine-Isosorbide Dinitrate’ OR ‘Hydralazine-Isosorbide NEAR/6 Dinitrate’ OR ‘Hydralazine Isosorbide Dinitrate’ OR ‘Hydralazine NEAR/6 Isosorbide NEAR/6 Dinitrate’ OR vasodilators):ab,ti,kw |
| 20 | Digoxin/exp or Digoxin:ab,ti,kw |
| 21 | ‘phosphodiesterase-5 inhibitor’/exp OR (sidenafil or tadalafil):ab,ti,kw |
| 22 | ‘sodium glucose cotransporter 2 inhibitor’/exp |
| 23 | (‘sodium-glucose co-transporter 2’ OR SGLT2 OR ‘SGLT2 inhibitor*’ OR ‘sodium glucose NEAR/6 inhibitor*’ OR ‘sodium-glucose NEAR/6 inhibitor*’ OR ‘Sodium-Glucose Transporter 2’ OR ‘sodium glucose-cotransporter 2’):ab,ti,kw |
| 24 | (dapagliflozin or empagliflozin or canagliflozin):ab,ti,kw |
| 25 | (‘diuretic agent’ OR ‘loop diuretic agent’)/exp |
| 26 | (furosemide or bumetanide or torasemide or azosemide or eplerenone or hydroclorotiazides):ab,ti,kw |
| 27 | or/6-26 |
| 28 | 27 AND [randomized controlled trial]/lim |
| 29 | 28 AND [English]/lim |
| 30 | 29 AND [human]/lim |

**Supplementary Table 3.** Search strategy in Cochrane Central Register of Controlled Trials (Date of search: Feb 22, 2022)

| # | Query |
| --- | --- |
| 1 | (“heart failure with preserved ejection fraction”[Mesh]) OR (“diastolic heart failure”[Mesh] ) OR (“heart failure with preserved ejection fraction”[Title/Abstract] ) OR (“diastolic heart failure”[Title/Abstract]) |
| 2 | (“randomized controlled trial”[Publication Type] OR (randomized[Publication Typet]) OR “randomized controlled trial”[Title/Abstract] OR randomization [Title/Abstract] |
| 3 | (“angiotensin receptor neprilysin inhibitor”[Mesh]) OR (“sacubitril–valsarta” [Mesh] ) OR (“LCZ696” [Mesh]) OR (“angiotensin receptor neprilysin inhibitor” [Title/Abstract]) OR (“sacubitril–valsarta”[Title/Abstract]) OR (“LCZ696” [Title/Abstract] ) |
| 4 | (“angiotensin converting enzyme inhibitor”[Mesh]) OR (“perindopril” [Mesh] ) OR (“quinapril” [Mesh] ) OR (“perindopri” [Mesh] ) OR (“benazepril” [Mesh] ) OR (“catopril” [Mesh] ) OR (“fosinopril” [Mesh] ) OR (“enalapril” [Mesh] ) OR (“ramipril” [Mesh] ) OR (“angiotensin converting enzyme inhibitor” [Title/Abstract]) OR (“perindopril” [Title/Abstract]) OR (“quinapril” [Title/Abstract] ) OR (“perindopri” [Title/Abstract] ) OR (“benazepril” [Title/Abstract] ) OR (“catopril” [Title/Abstract] ) OR (“Fosinopril” [Title/Abstract] ) OR (“Enalapril” [Title/Abstract] ) OR (“ramipril” [Title/Abstract] ) |
| 5 | (“angiotensin receptor blockers”[Mesh]) OR (“irbesartan” [Mesh] ) OR (“valsartan” [Mesh] ) OR (“candesartan” [Mesh]) OR (“losartan” [Mesh]) OR (“angiotensin receptor blockers”[Title/Abstract]) OR (“Irbesartan” [Title/Abstract] ) OR (“valsartan” [Title/Abstract]) OR (“candesartan” [Title/Abstract]) OR (“losartan” [Title/Abstract] ) |
| 6 | (“beta blockers”[Mesh]) OR (“propranolol” [Mesh] ) OR (“carvedilol” [Mesh] ) OR (“metoprolol” [Mesh]) OR (“bisoprolol” [Mesh]) OR (“beta blockers”[Title/Abstract]) OR (“propranolol”[Title/Abstract]) OR (“carvedilol”[Title/Abstract]) OR (“metoprolol” [Title/Abstract] ) OR (“bisoprolol” [Title/Abstract] ) |
| 7 | (“mineralocorticoid receptor antagonists”[Mesh]) OR (“spirolactone” [Mesh] ) OR (“eplerenone” [Mesh] ) OR (“antisterone” [Mesh]) OR (“aldactone” [Mesh]) OR “mineralocorticoid receptor antagonists”[Title/Abstract]) OR (“spirolactone” [Title/Abstract]) OR (“eplerenone”[Title/Abstract]) OR (“antisterone” [Title/Abstract] ) OR (“aldactone” [Title/Abstract] ) |
| 8 | (“digoxin”[Mesh]) OR (“digoxin” [Title/Abstract] ) |
| 9 | (“phosphodiesterase-5 inhibition”[Mesh]) OR (“sidenafi” [Mesh]) OR (“tadalafil” [Mesh]) OR (“phosphodiesterase-5 inhibition”[Title/Abstract]) OR (“sidenafi” [Title/Abstract]) OR (“tadalafil” [Title/Abstract] ) |
| 10 | (“soluble guanylate cyclase stimulator”[Mesh]) OR (“vericiguat” [Mesh]) OR (“soluble guanylate cyclase stimulator”[Title/Abstract]) OR (“vericiguat” [Title/Abstract]) |
| 11 | (“sodium-glucose cotransporter-2”[Mesh]) OR (“dapagliflozin”[Mesh]) OR (“ canagliflozin” [Mesh] ) OR (“sodium-glucose cotransporter-2”[Title/Abstract]) OR (“dapagliflozin” [Title/Abstract] ) OR (“ canagliflozin” [Title/Abstract] ) |
| 12 | OR/3-11 |
| 13 | 1 AND 2 AND 12 |

**Supplementary Table 4.** Summary of trial-specific definitions for the composite outcome of cardiovascular death and hospitalization for heart failure. HF, heart failure.

| **First author year (Trial Name)** | **Definitions** |
| --- | --- |
| Solomon 2022 (DELIVER) | Composite of worsening HF (unplanned hospitalization for HF or an urgent visit for HF), or cardiovascular death |
| Bhatt 2021 (SOLOIST-WHF) | Deaths from cardiovascular causes and hospitalizations and urgent visits for HF |
| Anker 2021 (EMPEROR-Preserved) | Composite of adjudicated cardiovascular death or hospitalization for HF |
| Packer 2020, Lam 2021 (EMPEROR-Reduced) | Composite of adjudicated cardiovascular death or hospitalization for HF |
| Armstrong 2020 (VICTORIA) | Composite of death from cardiovascular causes or first hospitalization for HF |
| McMurray 2019 (DAPA-HF) | Composite of worsening HF (unplanned hospitalization or an urgent visit resulting in intravenous therapy for HF) and death from cardiovascular causes |
| Solomon 2019 (PARAGON-HF) | Composite of total (first and recurrent) hospitalizations for HF and death from cardiovascular causes |
| Morrow 2019, Berardi 2020 (PIONEER-HF) | Cardiovascular mortality and HF rehospitalization were reported as clinical events by sites and later were adjudicated by a blinded clinical events expert |
| McMurray 2014 (PARADIGM-HF) | Composite of death from cardiovascular causes or a first hospitalization for HF |
| Gheorghiade 2013 (ASTRONAUT) | First occurrence of cardiovascular death or rehospitalization for HF at 6 months (ie, 190 days) after randomization |
| Konstam 2007 (EVEREST) | composite of cardiovascular mortality or cardiovascular hospitalization; incidence of cardiovascular mortality; and incidence of clinical worsening of HF (death, hospitalization for HF, or unscheduled visit for HF) |
| MERIT-HF Study group 1999, Goldstein 2003 (MERIT-HF) | All-cause mortality or hospitalization due to worsening HF |
| The SOLVD Investigators 1992, Dries 1999 (SOLVD) | death from any cause or hospitalization for HF |

HF, heart failure.

**Supplementary Table 5.** All studies included with risk of bias assessment

| **Author year (Study Name)** | **Random sequence generation** | **Allocation concealment** | **Blinding (participants and personnel)** | **Blinding (outcome assessment)** | **Selective reporting** | **Incomplete outcome data** | **Other bias** |
| --- | --- | --- | --- | --- | --- | --- | --- |
| Solomon 2022 (DELIVER)^1^ | Low | Low | Low | Low | Low | Low | Low |
| Bhatt 2021 (SOLOIST-WHF)^2^ | Low | Low | Low | Low | Low | High | Low |
| Anker 2021 (EMPEROR-Preserved)^3^ | Low | Low | Low | Low | Low | Low | Low |
| Packer 2020, Lam 2021 (EMPEROR-Reduced)^4,5^ | Low | Low | Low | Low | Low | Low | Low |
| Armstrong 2020 (VICTORIA)^6^ | Low | Unclear | Low | Low | Low | Low | Low |
| McMurray 2019 (DAPA-HF)^7^ | Low | Low | Low | Low | Low | Low | Low |
| Solomon 2019 (PARAGON-HF)^8^ | Low | Low | Low | Low | Low | Low | Low |
| Morrow 2019, Berardi 2020 (PIONEER-HF)^9,10^ | Unclear | Unclear | Unclear | Low | Low | Low | Unclear |
| McMurray 2014 (PARADIGM-HF)^11^ | Low | Low | Low | Low | Low | Low | Low |
| Gheorghiade 2013 (ASTRONAUT)^12^ | Low | Low | Low | Low | Low | Low | Low |
| Konstam 2007 (EVEREST)^13^ | Low | Unclear | Low | Low | Low | Low | Low |
| BEST investigators, 2001 (BEST)^14^ | Low | Low | Low | Low | Low | Low | Low |
| Levine 2000 (MACH-1)^15^ | Low | Unclear | Low | Low | Unclear | Unclear | Low |
| MERIT-HF Study group 1999, Goldstein 2003 (MERIT-HF)^16,17^ | Low | Low | Low | Low | Low | Unclear | Low |
| Packer 1996, Yancy 2001 (U.S. Carvedilol HF)^18,19^ | Low | Low | Low | Low | Unclear | Low | High |
| Pfeffer 1992, Moye 1994 (SAVE)^20,21^ | Low | Unclear | Low | Low | Unclear | Low | Low |
| The SOLVD Investigators 1992, Dries 1999 (SOLVD)^22,23^ | Unclear | Unclear | Low | Low | Low | Low | Low |
| Cohn 1991, Carson 1999 (V-HeFT II)^24,25^ | Low | Unclear | High | Low | Unclear | Low | Low |
| Cohn 1986, Carson 1999 (V-HeFT)^25,26^ | Low | Unclear | Low | Low | Unclear | Low | Low |

**Supplementary Figure 1** The PRISMA flowchart of trial selection. CENTRAL, Cochrane Central Register of Controlled Trials


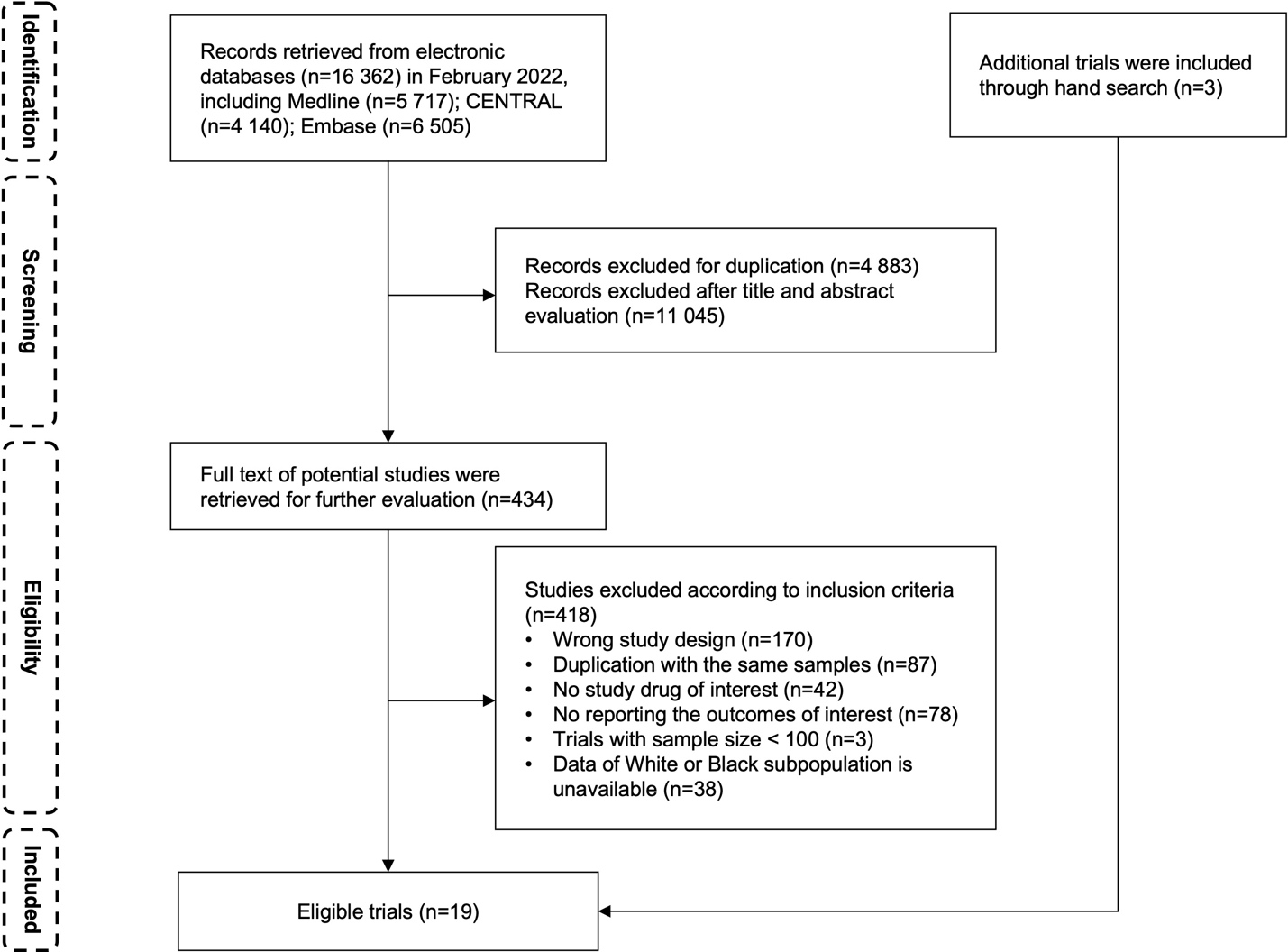


**Supplementary Figure 2.** Forest plot showing pooled risk ratios for different pharmacologic therapies among White participants for (A) all-cause death, (B) HHF, and (C) composite of CV death or HHF. RR, relative risk; CI, confidence interval; ACEi, angiotensin-converting enzyme inhibitor; HHF, hospitalization for heart failure; CV, cardiovascular; SGLT2i, sodium–glucose cotransporter 2 inhibitor; ARNI, angiotensin receptor–neprilysin inhibitor.

**A.** All-cause mortality by drug class


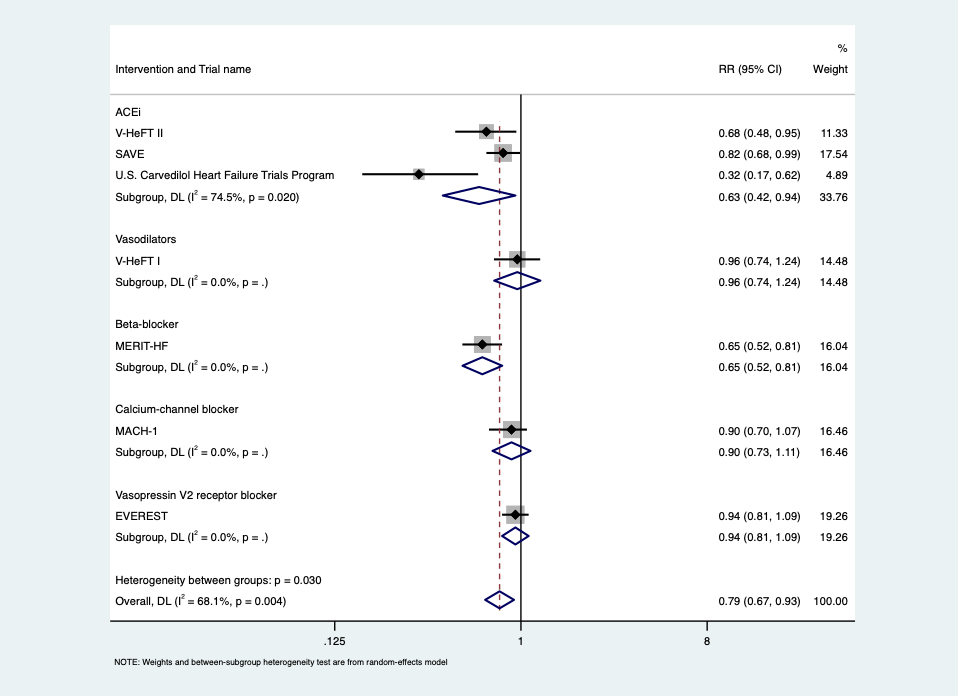


**B.** HHF hospitalization by drug class


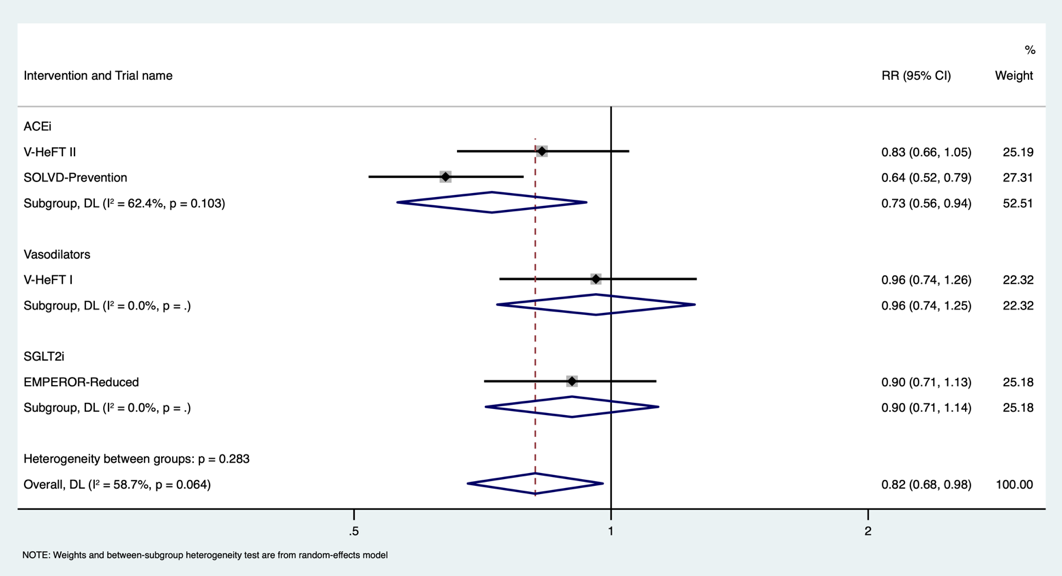


**C.** CV death or HHF by drug class


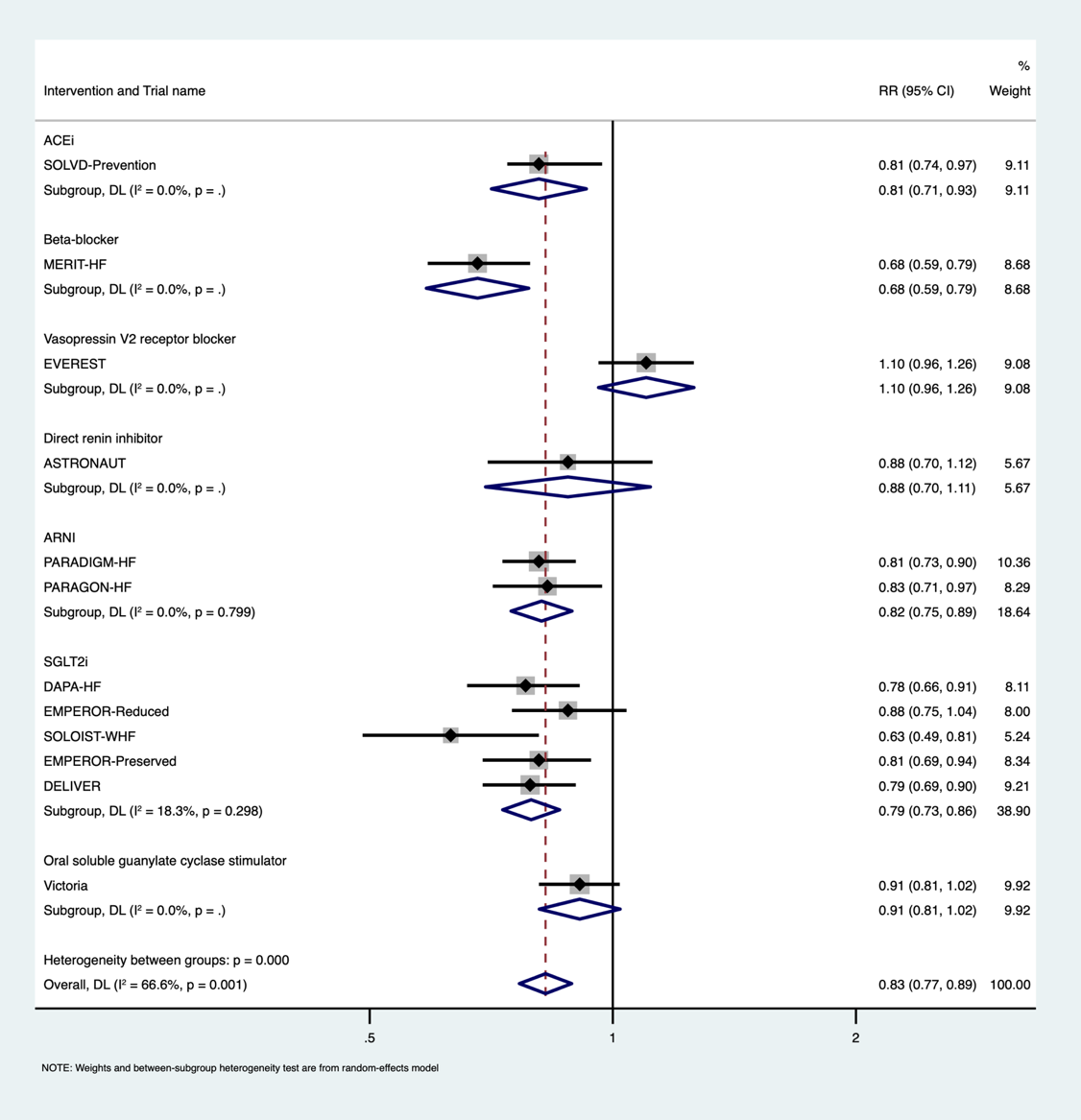


**Supplementary Figure 3.** Forest plot showing pooled risk ratios for different pharmacologic therapies among Black participants for (A) all-cause death, (B) HHF, and (C) composite of CV death or HHF. RR, relative risk; CI, confidence interval; ACEi, angiotensin-converting enzyme inhibitor; HHF, hospitalization for heart failure; CV, cardiovascular; SGLT2i, sodium–glucose cotransporter 2 inhibitor; ARNI, angiotensin receptor–neprilysin inhibitor;

1. All-cause mortality by drug class


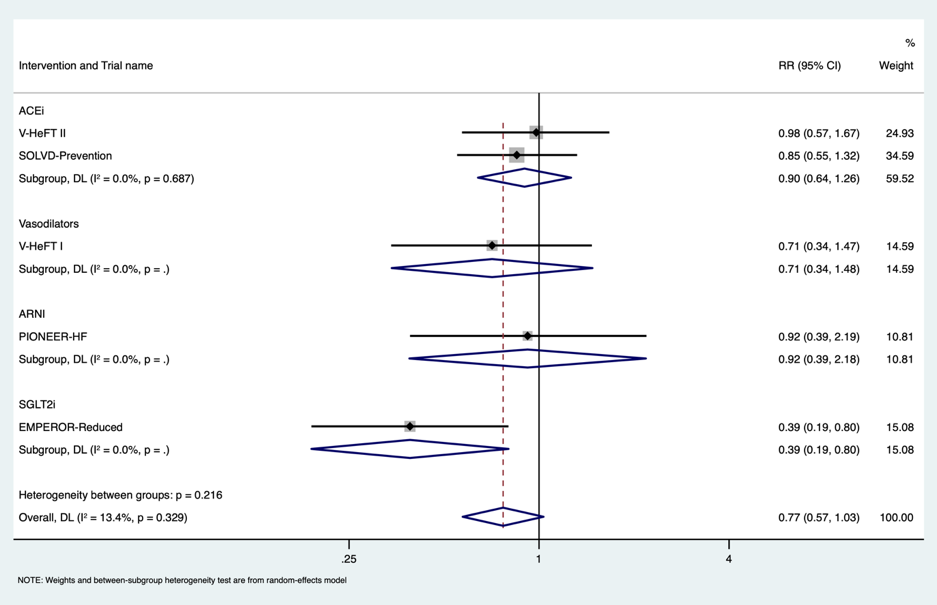


**B.** HHF hospitalization by drug class


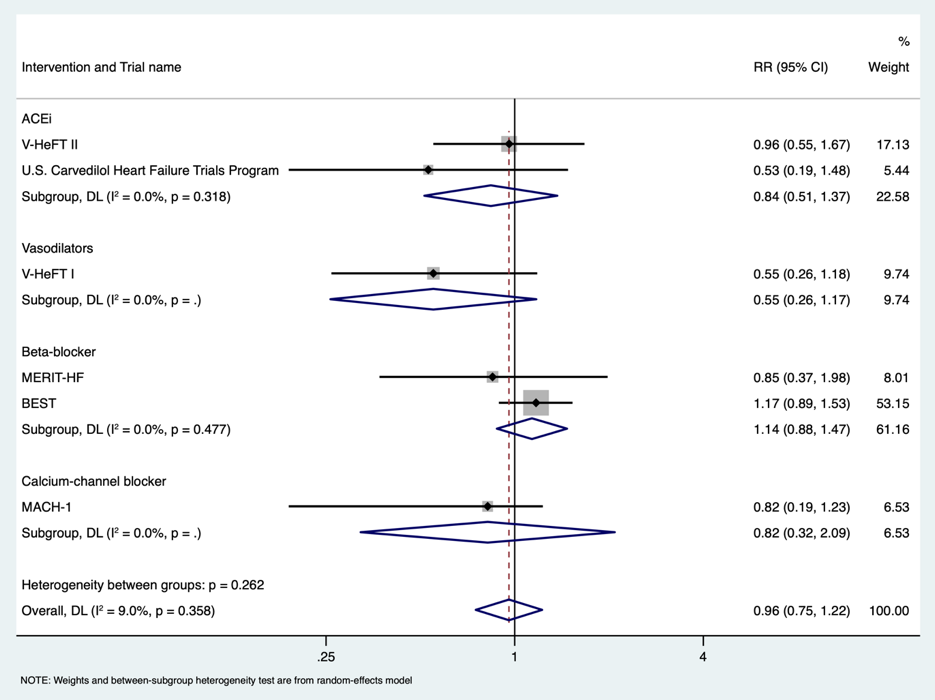
**C.** CV death or HHF by drug class


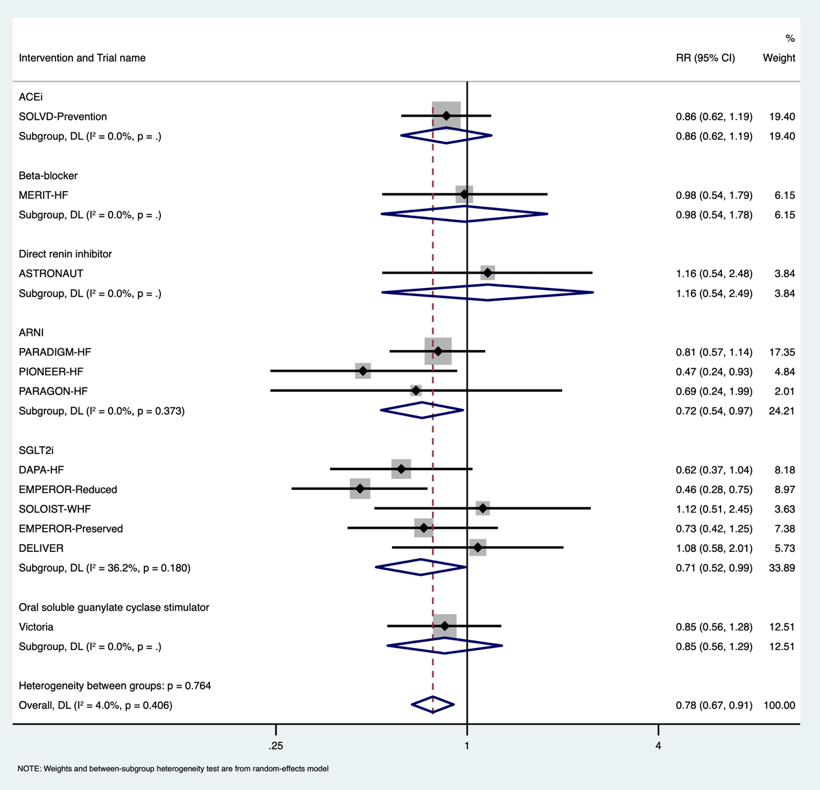


**Reference**

1. Solomon SD, McMurray JJV, Claggett B, et al. Dapagliflozin in Heart Failure with Mildly Reduced or Preserved Ejection Fraction. *N Engl J Med*. 2022;387(12):1089-1098.

2. Bhatt DL, Szarek M, Steg PG, et al. Sotagliflozin in Patients with Diabetes and Recent Worsening Heart Failure. *N Engl J Med*. 2021;384(2):117-128.

3. Anker SD, Butler J, Filippatos G, et al. Empagliflozin in Heart Failure with a Preserved Ejection Fraction. *N Engl J Med*. 2021;385(16):1451-1461.

4. Packer M, Anker SD, Butler J, et al. Cardiovascular and Renal Outcomes with Empagliflozin in Heart Failure. *N Engl J Med*. 2020;383(15):1413-1424.

5. Lam CSP, Ferreira JP, Pfarr E, et al. Regional and ethnic influences on the response to empagliflozin in patients with heart failure and a reduced ejection fraction: the EMPEROR-Reduced trial. *Eur Heart J*. 2021;42(43):4442-4451.

6. Armstrong PW, Pieske B, Anstrom KJ, et al. Vericiguat in Patients with Heart Failure and Reduced Ejection Fraction. *N Engl J Med*. 2020;382(20):1883-1893.

7. McMurray JJV, Solomon SD, Inzucchi SE, et al. Dapagliflozin in Patients with Heart Failure and Reduced Ejection Fraction. *N Engl J Med*. 2019;381(21):1995-2008.

8. Solomon SD, McMurray JJV, Anand IS, et al. Angiotensin–Neprilysin Inhibition in Heart Failure with Preserved Ejection Fraction. *N Engl J Med*. 2019;381(17):1609-1620.

9. Morrow DA, Velazquez EJ, DeVore AD, et al. Clinical Outcomes in Patients With Acute Decompensated Heart Failure Randomly Assigned to Sacubitril/Valsartan or Enalapril in the PIONEER-HF Trial. *Circulation*. 2019;139(19):2285-2288.

10. Berardi C, Braunwald E, Morrow DA, et al. Angiotensin-Neprilysin Inhibition in Black Americans. *JACC Heart Fail*. 2020;8(10):859-866.

11. McMurray JJV, Packer M, Desai AS, et al. Angiotensin–Neprilysin Inhibition versus Enalapril in Heart Failure. *N Engl J Med*. 2014;371(11):993-1004.

12. Gheorghiade M, Böhm M, Greene SJ, et al. Effect of Aliskiren on Postdischarge Mortality and Heart Failure Readmissions Among Patients Hospitalized for Heart Failure: The ASTRONAUT Randomized Trial. *JAMA*. 2013;309(11):1125-1135.

13. Konstam MA, Gheorghiade M, Burnett JC, et al. Effects of oral tolvaptan in patients hospitalized for worsening heart failure: the EVEREST Outcome Trial. *JAMA*. 2007;297(12):1319-1331.

14. The Beta-Blocker Evaluation of Survival Trial Investigators. A Trial of the Beta-Blocker Bucindolol in Patients with Advanced Chronic Heart Failure. *N Engl J Med*. 2001;344(22):1659-1667.

15. Levine TB, Bernink PJLM, Caspi A, et al. Effect of Mibefradil, a T-Type Calcium Channel Blocker, on Morbidity and Mortality in Moderate to Severe Congestive Heart Failure. *Circulation*. 2000;101(7):758-764.

16. MERIT-HF Study Group. Effect of metoprolol CR/XL in chronic heart failure: Metoprolol CR/XL Randomised Intervention Trial in-Congestive Heart Failure (MERIT-HF). *The Lancet*. 1999;353(9169):2001-2007.

17. Goldstein S, Deedwania P, Gottlieb S, Wikstrand J. *Metoprolol* CR/XL in black patients with heart failure (from the *Metoprolol* CR/XL randomized intervention trial in chronic heart failure). *Am J Cardiol*. 2003;92(4):478-480.

18. Packer M, Bristow MR, Cohn JN, et al. The effect of carvedilol on morbidity and mortality in patients with chronic heart failure. U.S. Carvedilol Heart Failure Study Group. *N Engl J Med*. 1996;334(21):1349-1355.

19. Yancy CW, Fowler MB, Colucci WS, et al. Race and the response to adrenergic blockade with carvedilol in patients with chronic heart failure. *N Engl J Med*. 2001;344(18):1358-1365.

20. Moyé LA, Pfeffer MA, Wun CC, et al. Uniformity of captopril benefit in the SAVE study: subgroup analysis. *Eur Heart J*. 1994;15(suppl_B):2-8.

21. Pfeffer MA, Braunwald E, Moyé LA, et al. Effect of Captopril on Mortality and Morbidity in Patients with Left Ventricular Dysfunction after Myocardial Infarction. *N Engl J Med*. 1992;327(10):669-677.

22. The SOLVD Investigators. Effect of Enalapril on Mortality and the Development of Heart Failure in Asymptomatic Patients with Reduced Left Ventricular Ejection Fractions. *N Engl J Med*. 1992;327(10):685-691.

23. Dries DL, Exner DV, Gersh BJ, Cooper HA, Carson PE, Domanski MJ. Racial Differences in the Outcome of Left Ventricular Dysfunction. *N Engl J Med*. 1999;340(8):609-616.

24. Cohn JN, Johnson G, Ziesche S, et al. A Comparison of Enalapril with Hydralazine–Isosorbide Dinitrate in the Treatment of Chronic Congestive Heart Failure. *N Engl J Med*. 1991;325(5):303-310.

25. Carson P, Ziesche S, Johnson G, Cohn JN. Racial differences in response to therapy for heart failure: analysis of the vasodilator-heart failure trials. Vasodilator-Heart Failure Trial Study Group. *J Card Fail*. 1999;5(3):178-187.

26. Cohn JN, Archibald DG, Ziesche S, et al. Effect of vasodilator therapy on mortality in chronic congestive heart failure. Results of a Veterans Administration Cooperative Study. *N Engl J Med*. 1986;314(24):1547-1552.
